# Defeat Such Legislation

**Published:** 1901-03

**Authors:** 


### DEFEAT SUCH LEGISLATION.

Senate bill No. 51, of the Pennsylvania State Legislature, to reopen registrations to non-graduates, as provided for in the law of 1889 and its supplement of 1891, should receive an overwhelming defeat at the hands of the Legislature through the efforts of the veterinary profession in the Keystone State. The Legislative Committee of the State Association are thoroughly aroused, and with more than eight hundred active veterinarians in the State there should be no rest until the bill is scotched. Every veterinarian should be at his post of duty until its defeat is assured.

On another page will be found the announcement of the death of Professor Williams, of Edinburgh, Scotland. Though having reached nearly three-score and ten, his death was sudden and unexpected. Known wherever the English language is read or spoken as a voluminous contributor to veterinary literature, both as a journalist and in the preparation of books that have for years been accepted as standard ones, he had attained a world-wide reputation. As a teacher and chief director of one of the leading English veterinary colleges he had done valuable work and reared a worthy monument to his name. For the people of his own country he had performed valuable services in special investigations of diseases of livestock.

